# On the Treatment of Pneumonia

**Published:** 1847-05

**Authors:** Anthony Todd Thompson


*On the Treatment of Pneumonia.*—By Dr. ANTHONY TODD THOMPSON. The writer's object in the following remarks is to examine the comparative advantages of treating pneumonia with bleeding, and with antimony, with or without mercury. In regard to the former, while he acknowledges its utility when employed with moderation and at an early period of the disease, he deprecates profuse and repeated bleedings as positively hurtful. He denies that it is ever beneficial when solidification of the lungs has taken place, but on the contrary, asserts that it only quickens the circulation, and hastens the disorganizing process. He declares his conviction that the repeated use of the lancet may generally be rendered unnecessary by the judicious employment of tartar emetic. His practice is thus developed:

My practice, as soon as I have fully satisfied myself of the existence of the disease, and if the attack has not run on to the second stage, is to order one bleeding to the amount of sixteen or twenty ounces; to follow this immediately with three or four grains of calomel and one grain of opium, with the view of preventing that nervous irritability which often succeeds the use of the lancet, and of sustaining the beneficial impression made on the system by blood-letting. In two hours afterwards I give one grain of potassio-tartrate of antimony, in a fluid ounce and a half of emulsion of bitter almonds, and repeat this dose every third or fourth hour, until a decided diminution of inflammatory action takes place—that is, until the crepitation has nearly disappeared, and the sputa are no longer rusty and tenacious. The intervals between the doses of the tartar emetic are then extended to six hours, and afterwards to eight hours, and so continued until convalescence is confirmed. I prefer the bitter almond emulsion on account of its containing hydrocyanic acid, which has a sedative quality, and a more decided influence in quieting the nervous system, and abating the cough, than small doses of opium. When pneumonia is uncomplicated, this plan, with the occasional aid of some mild aperient, has, in my hands, seldom failed to carry the case to a successful termination. When the attack has passed the first stage, when dullness on percussion indicates hepatization, then the object of the second indication—namely, to excite the capillaries and prevent further depositions—requires attention; and, in order to fulfil this indication, I order four or five grains of mercury with chalk, or one grain of calomel, to be given in each interval of the administration of the tartar emetic. In the statistical table, twenty-three out of the thirty-eight cases were treated with mercurials in addition to the tartar emetic, because, in hospital practice, the patients are seldom brought in until the disease has progressed, and the solid effusion, if I may employ such a term, has taken place. But in the remaining fifteen cases no mercurial was employed, the tartar emetic was alone trusted to, and it did not disappoint my anticipations; but in no instance was its employment commenced until after the abstraction of blood, either by the lancet or by cupping. I have never carried the mercurial action to ptyalism; but it is requisite to procure the usual symptoms of its decided impression on the habit, by producing slight tenderness of the gums. In ordinary cases I have seldom had occasion to order blisters; but, from the nature of hospital practice, they are demanded, and they were employed in sixteen of the cases in the statistical table. Such are the advantages of moderate doses of tartar emetic in the treatment of pneumonia. In the same manner as when larger doses are administered, vomiting almost always occurs after the first and the second dose of the remedy; but on the third dose the tolerance is established, and even when the dose is increased, neither nausea nor vomiting afterwards takes place.

In reference to the *modus operandi* of tartar emetic, the author further remarks:

If I may be permitted to state a hypothetical opinion, I should venture to offer my view of what has been termed the tolerance of the habit against the well known influence of tartar emetic, when administered in moderately large doses. It has been supposed by Rasori and others, that the inflammatory state of the system in pneumonia is the cause of this tolerance, and that this would cease if the remedy were continued after every trace of general inflammation had disappeared. I am, however, of opinion, that the tolerance does not depend on the state of the habit, but solely on the topical influence of the tartar emetic on the mucous membrane; and upon its influence, also, on the membrane operating as a counter-irritant; and to this its beneficial effect in subduing inflammation is to be attributed. When a grain or more of tartar emetic is swallowed, a portion is carried into the circulation, and acting on the nervous centres, it causes vomiting; but a portion also acts topically, on the mucous membrane, and sets up subacute inflammatory action there. The second dose also causes vomiting, because the inflammation, although extended, yet is not sufficient to prevent absorption: but when the third dose is given, the absorption no longer takes place, and consequently no vomiting is induced. It is a well-established fact, that an inflamed surface is incompatible with absorption: and to this, in a great measure, are we to attribute the accumulation of serum in serous cavities in dropsy. If my views be admitted, I would further state, that the determination of blood to so extended a surface as that of the mucous membrane is, in my opinion, sufficient to relieve the congestion in the lungs; and that it is, in truth, the cause of the benefit which follows the employment of the tartar emetic. Such is my opinion of the rationale of the action of tartar emetic in pneumonia; but on this part of my subject it is not my intention to insist, my chief object being to demonstrate that this powerful therapeutical agent can perform all that can be expected from its administration in much smaller doses than Rasori and his followers have employed; that such doses incur no risk, seldom bringing on diarrhœa, whilst hypercatharsis has followed the administration of larger doses; and that in such doses it sets aside the necessity of large and repeated abstractions of blood, and consequently cures the disease without that injurious impression on the habit which has been frequently seen to follow copious blood-letting. Another advantage obtained by this mode of practice in pneumonia is the gradual advancement of the convalescence, and the restoration of the patient to health and vigor without the aid of tonics. By gradually letting down the action of the tartar emetic, the stomach recovers its tone, and the assimilating function rapidly restores any waste which the necessary treatment of the disease may have occasioned.—*London Lancet.*—*Ibid.*

